# Swim-Training Changes the Spatio-Temporal Dynamics of Skeletogenesis in Zebrafish Larvae (*Danio rerio*)

**DOI:** 10.1371/journal.pone.0034072

**Published:** 2012-04-18

**Authors:** Ansa W. Fiaz, Karen M. Léon-Kloosterziel, Gerrit Gort, Stefan Schulte-Merker, Johan L. van Leeuwen, Sander Kranenbarg

**Affiliations:** 1 Experimental Zoology Group, Department of Animal Sciences, Wageningen University and Research Centre, Wageningen, The Netherlands; 2 Biometris, Wageningen University and Research Centre, Wageningen, The Netherlands; 3 Hubrecht Institute, KNAW and UMC Utrecht, Utrecht, The Netherlands; Universitat de Barcelona, Spain

## Abstract

Fish larvae experience many environmental challenges during development such as variation in water velocity, food availability and predation. The rapid development of structures involved in feeding, respiration and swimming increases the chance of survival. It has been hypothesized that mechanical loading induced by muscle forces plays a role in prioritizing the development of these structures. Mechanical loading by muscle forces has been shown to affect larval and embryonic bone development in vertebrates, but these investigations were limited to the appendicular skeleton. To explore the role of mechanical load during chondrogenesis and osteogenesis of the cranial, axial and appendicular skeleton, we subjected zebrafish larvae to swim-training, which increases physical exercise levels and presumably also mechanical loads, from 5 until 14 days post fertilization. Here we show that an increased swimming activity accelerated growth, chondrogenesis and osteogenesis during larval development in zebrafish. Interestingly, swim-training accelerated both perichondral and intramembranous ossification. Furthermore, swim-training prioritized the formation of cartilage and bone structures in the head and tail region as well as the formation of elements in the anal and dorsal fins. This suggests that an increased swimming activity prioritized the development of structures which play an important role in swimming and thereby increasing the chance of survival in an environment where water velocity increases. Our study is the first to show that already during early zebrafish larval development, skeletal tissue in the cranial, axial and appendicular skeleton is competent to respond to swim-training due to increased water velocities. It demonstrates that changes in water flow conditions can result into significant spatio-temporal changes in skeletogenesis.

## Introduction

Fish larvae are the smallest free-living vertebrates. They encounter many environmental challenges during development such as variation in water velocity, food availability and predation. Osse and van den Boogaart [1] hypothesized that developmental patterns of form change and growth in larval fish which optimize survival chances are maintained during the course of evolution. Moreover, Fuiman [2] and Osse and van den Boogaart [1] showed that growth of the head and tail area were prioritized over the growth of the trunk area during fish larval development. In fish larvae, the first skeletal elements to develop are structures involved in feeding (e.g. cleithrum, 5th branchial arch), respiration (e.g. opercular bones) and swimming (e.g. hypurals) [3]–[6]. The rapid development of structures involved in feeding and swimming is important for survival on exogenous food and to escape predation.

Weisel [3] investigated early bone development in the sucker *Catostomus macrocheilus* and found that the bones which formed earliest were associated with movement such as feeding, respiration and swimming rather than protection. This suggests that mechanical loading induced by muscle forces plays a role in prioritizing the development of these structures.

Mechanical loading by muscle forces has been shown to affect embryonic and larval bone development in vertebrates, but these investigations have been limited to the appendicular skeleton. In humans, Rodríguez *et al.* [7] demonstrated in a radiographic and histological study that fetal immobility led to thin, hypomineralized, and slender long bones. In avian and murine embryos, *in vivo* experiments with decreased mechanical loading led to a reduction in mineralization, bone surface area, chondrocyte proliferation and glycosaminglycan content of cartilage in long bone rudiments [8]–[12]. Increased *in vivo* mechanical loading led to longer leg bones in chick embryos [13]–[14]. In addition, *in vitro* experiments showed that increased mechanical loading increased the rate of bone and cartilage formation in chick bone rudiments and calcification of the cartilage growth plate in mouse long bone rudiments [15]–[16]. In fish, Cloutier *et al.* [17] showed that swim-training accelerated the onset of chondrogenesis and osteogenesis in the median fins in the Artic Charr (*Salvelinus alpinus*). Previous work from our lab showed that swim-training accelerated osteogenesis of the tail bones during late larval and juvenile development in zebrafish [18]. Analysis of the tail region showed that the onset of osteogenesis of the hypurals and spines of the caudal peduncle appeared earlier in the trained fish than in the control fish.

To investigate the role of mechanical loading in prioritizing the development of structures involved in feeding, respiration and swimming, we analyzed the effect of swim-training during early skeletal development in zebrafish. It is likely that swim-training increases mechanical loading via increased muscle activity. The *ex utero* development and the relatively late skeletal development of the zebrafish makes it possible to investigate the effect of swim-training on the onset of chondrogenesis and osteogenesis *in vivo*. We investigated the time of appearance of cartilage and bone structures in the cranial, axial and appendicular skeleton. In zebrafish embryos, coordinated patterns of swimming appear before the development of many skeletal elements. The first movements appear around 17–19 hours post-fertilization (hpf, [19]) as side-to-side contractions of the trunk and precede coordinated patterns of swimming behaviour. The onset of chondrification and ossification of many skeletal elements lies between 5–14 dpf [6] [5]. The transition from a notochord to a vertebral column also takes place in this period. Therefore, zebrafish larvae were subjected to swim-training from 5–14 dpf. From 72 hpf until 29 dpf (approximately 3.5 mm–10 mm total length), zebrafish are staged as larva [20]–[21].

Our study is the first one to investigate the effect of increased swimming activity on osteogenesis and chondrogenesis of the cranial, axial and appendicular skeleton during early fish larval development. Our study shows that increased swimming activity increases growth in zebrafish larvae and prioritizes the formation of skeletal elements that are involved in feeding, respiration and swimming. This reveals a previously unknown plasticity in early vertebrate chondrogenesis and osteogenesis of the cranial, axial and appendicular skeleton in response to an increased water velocity [17] [22]–[25].

## Results

### Swim Behaviour and Growth During Swim-training

Zebrafish larvae were subjected to swim-training for 9 hours per day from 5–14 dpf. Trained fish were kept in tubes in a custom built swim-training setup and subjected to 50% of the critical flow velocity (see also Materials and Methods). Control fish were kept in identical tubes in the same swim-training setup but with a continuous water flow of less than 0.1 Bl/s for refreshment. During the swim-training we observed the swimming behaviour of control and trained fish (see also Movies S1 and S2 of the swimming behaviour of control and trained fish). Until 12 dpf more than 80% of the trained fish swam along the wall of the tube. At 13 dpf this decreased to 76% and at 14 dpf further to 42%. At these stages, trained fish swam more in the central part of the tube. The number of trained fish swimming in the front part of the tubes (closer to the inflow of water) also decreased at 13 and 14 dpf. Until 12 dpf, on average, 47% of the trained fish swam in the front part of the tube, at 13 dpf this was 36% and at 14 dpf 13%. In the younger stages, trained fish adhered to the wall of the tube for more than 3 seconds (7%–1% from 5–8 dpf, respectively) to maintain position. Trained fish older than 8 dpf did not show this behaviour. During the swim-training, only occasionally did trained fish stick for more than 3 seconds against the mesh (<1%, 6 and 10 dpf). The control fish swam in all directions throughout the tube.

**Figure 1:**
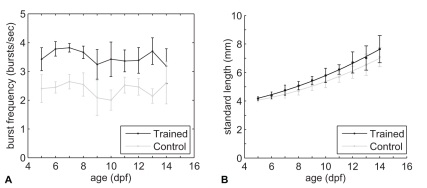
Swim-training increased burst frequency and growth in trained fish. **A**) Average number of bursts per second ± standard deviation as a function of age in days post fertilization (dpf). **B**) Average standard lengths (dots) ± standard deviation (with quadratic regression fit) as a function of age (dpf).

Both trained and control fish showed intermittent swim behaviour; short bursts of tail beats alternated with coasting. To investigate if trained fish had a higher burst frequency than control fish, we analyzed the number of bursts per second. Note that one burst represents several tail beats. Statistical analysis of the burst frequency showed that no relationship with age could be found (Figure 1A, ANCOVA, P>0.05). However, trained fish had on average a higher burst frequency (3.5 bursts/s) during the swim-training than control fish (2.5 bursts/s, t-test, P<0.05). During the breaks trained fish swam within 5 minutes throughout the tube like the control fish, indicating that trained fish recovered quickly from the training. Mortality ranged between 2–40% and was not significantly different between control and trained fish which indicates that swim-training did not influence survival.

Swim-training increased growth in trained fish compared to control fish (Figure 1B), as shown by quadratic regression of the standard length measurements on age in the two groups. A significant difference (P<0.05) between the coefficients of the linear terms for trained and control fishes was found. The curvature was not significantly different between the two groups, nor were the intercepts. These results indicate that length differences between trained and control groups increased linearly with age.

### Effect of Swim-training on Chondrogenesis

**Figure 2:**
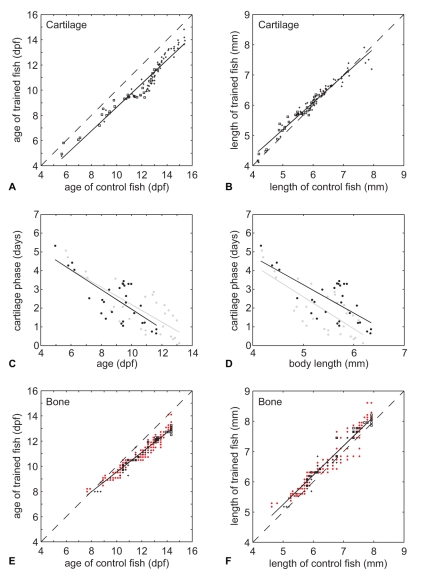
Swim-training accelerated both chondrogenesis and osteogenesis. **A**) CF50*_age_* values of trained fish plotted against CF50*_age_* values of control fish with a linear regression fit (solid line). Dashed line is a reference line which indicates the CF50*_age_* values which are not different between control and trained fish. Plus signs indicate cartilage structures without BF50*_age_* values, squares are with BF50*_age_* values. **B**) Similar plot as in A but with CF50*_lgt_* values. **C**) Duration of cartilage phase for cartilage bones (in days, indicated with squares in A and E) plotted against the CF50*_age_* values of control and trained fish with a linear regression fit (solid lines) (trained fish: black dots and black regression line, control fish: grey dots with grey regression line). **D**) Similar plot as in C but with duration of cartilage phase (days) plotted against the CF50*_lgt_* values of control and trained fish. **E**) Similar plot as in A but with BF50*_age_* values. Red dots indicates BF50*_age_* values of dermal bones, plus signs of cartilage bones without CF50*_age_* values, squares of cartilage bones with CF50*_age_* values. Dashed line see A. **F**) Similar plot as in E but with BF50*_lgt_* values.

The onset of chondrogenesis for each cartilage structure (trained and control fish) was estimated by the age or body length at which 50% of the fish showed the presence of a specific cartilage structure. We refer to the estimates of age as CF50*_age_* (Cartilage Forming) and to the estimates of body length as CF50*_lgt_*. CF50*_age_* and CF50*_lgt_* values were calculated from logistic regressions of the binary presence or absence data of cartilage structures (see also Materials and Methods). Plotting the CF50*_age_* values of trained fish against the CF50*_age_* values of control fish showed that swim-training accelerated chondrogenesis (Figure 2A, P<0.0001). Almost all of the CF50*_age_* values were located below the reference line, which indicates that cartilage structures appeared earlier in trained fish. However, plotting the CF50*_lgt_* values of trained fish against the CF50*_lgt_* values of control fish showed that trained fish were slightly longer at the onset of chondrogenesis during early development (Figure 2B, P<0.0001). This difference in CF50_lgt_ values between trained and control fish decreased with increasing length (P<0.0001).

In teleosts, bones can form via intramembranous or perichondral ossification. During intramembranous ossification, bones form directly from mesenchymal precursors whereas during perichondral ossification, bony matrix forms around a cartilage model [6]. We refer to the phase from the onset of chondrogenesis until the onset of osteogenesis as the cartilage phase. The duration of the cartilage phase in cartilage structures was calculated by subtracting the CF50*_age_* values from the BF50*_age_* values in control and trained fish. Plotting the duration of the cartilage phase (in days) against the CF50*_age_* and CF50*_lgt_* values showed that the duration of the cartilage phase decreases with increasing age and length, both in control and trained fish (Figure 2C and D, P<0.05).

**Figure 3:**
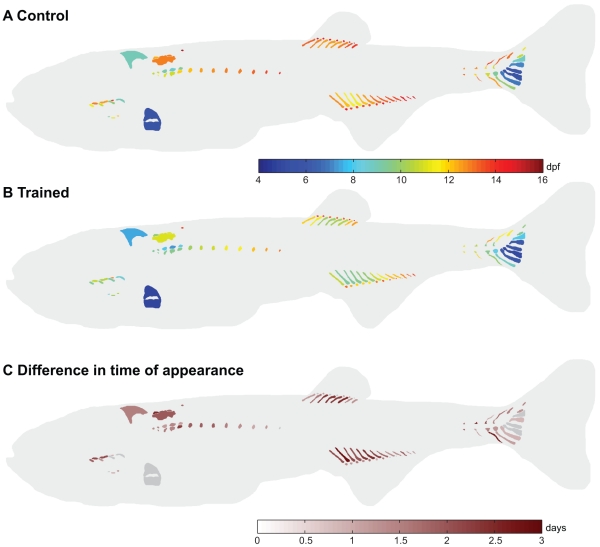
Swim-training had a differential effect on the age at appearance of cartilage structures between control and trained fish. **A,B**) CF50*_age_* values visualized in the corresponding structures in control fish (A) and trained fish (B). **C**) Differences in CF50*_age_* values between control and trained fish. Positive values indicate that structures appear earlier in the trained fish. Structures with a difference less than twice the standard error are indicated in grey.

In the control fish, some structures already appear before 5 dpf (e.g. Meckel’s cartilage, ceratobrancials 1–5, basihyal and hyosympletic (Figure S1)) and the CF50*_age_* values of these structures could therefore not be calculated from our data. The cartilage structures to appear between 5–8 dpf were the parhypural, hypural 1–3 and the pectoral fin endoskeleton (Figure 3A and Figure S1). Chondrogenesis proceeded with the appearance of the neural arches 3–5, epibranchials (ep) 3–4, hypobranchial (hb) 3, hypural 4, hemal arch and spine of preural vertebra (pu) 2 and the posterior part of the exoccipital between 8–10 dpf. The first structures of the Weberian apparatus and the first anal proximal radials (apr) 3–4 appeared between 10–12 dpf. Other cartilage structures which also appeared between 10–12 dpf were the ep 1–2, basibranchial 4, parapophyses (pop) 5, hypural 5, haemal arch 28, neural arch of pu 2–3 and haemal arch of pu 3. The paraphophyses appeared in an anterior to posterior direction. The proximal and distal radials of the anal and dorsal fin, however, appeared anteriorly and posteriorly to the third and fourth radial. The following cartilage structures appeared between 12–14 dpf: hb 4, pharyngobranchials (pb) 1–4, supraneural 2–3, roofing cartilage, parapophyses 6–12, apr 1–2, 5–10, anal distal radials (adr) 2–6 (with adr 4 as the first), dorsal proximal radials (dpr) 1–8, neural arch 27–28, haemal spine 28, neural spine of pu2 and neural and haemal spine of pu3. The cartilage structures to appear at 14 dpf and later were pop 13, supraneural 5, apr 11–13, adr 1, 7–10 and dorsal distal radials (ddr) 1–7.

Analysis of the difference in the CF50*_age_* values (temporal shift) between control and trained fish showed that swim-training had a differential effect on the temporal shift of cartilage structures (Figure 3C). The average standard error of the CF50*_age_* values was slightly higher in the control fish (0.34 days) than in trained fish (0.31 days). The minimum and maximum standard errors were also higher in the control fish than in trained fish (control: min 0.29 days, max 0.66 days; trained: min 0.29 days, max 0.52 days). Of the 92 cartilage elements analyzed in total, 77 cartilage elements showed a temporal shift which was larger than 2x SED (standard error of the difference). The minimum temporal shift of these 77 elements was 0.9 days and the maximum was 2.8 days. Chondrogenesis in these elements occurred on average 1.6 days earlier. In the cranial skeleton (11 elements examined in total), 8 elements had a temporal shift larger than 2x SED such as the epibranchials 1–2. In the axial skeleton (26 elements examined in total), 25 elements had a temporal shift larger than 2x SED such as the Weberian apparatus (e.g. tripus, supraneurals 2,3,5) and parapophyses 5–12. In the appendicular skeleton (55 elements examined in total), 44 elements had a temporal shift larger than 2x SED such as the anal and dorsal radials and cartilage structures in the caudal peduncle (e.g. epural, haemal and neural spines and arches of pu 2 and 3).

**Figure 4:**
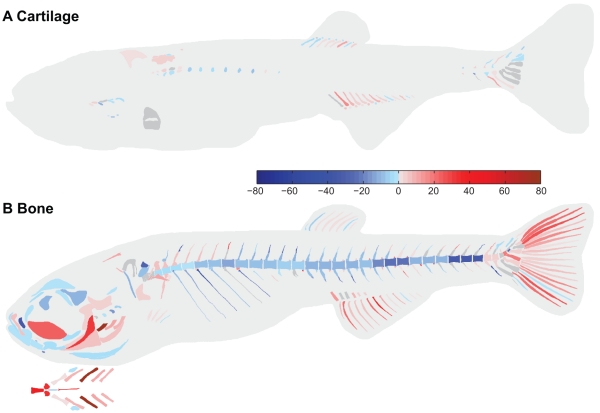
Swim-training had a differential effect on the order of appearance of cartilage and bone structures between control and trained fish. **A,B**) Difference in the rank of cartilage (A) and bone (B) structures between control and trained fish. Red structures indicate a forward shift in the order of appearance, blue structures a delay. Structures which did not show a difference are indicated in grey.

Analysis of the difference in order of appearance (rank shift, based on CF50*_age_*) of cartilage structures between control and trained fish showed that swim-training had also a differential effect on the rank shift of cartilage structures (Figure 4A). Of the 92 cartilage elements analyzed in total, 42 elements showed a forward rank shift in the order of appearance in the trained fish versus the control fish. In the cranial skeleton (11 elements examined in total), 3 elements showed a forward rank shift such as epibranchial 1. In the axial skeleton (26 elements examined in total), 11 elements showed a forward rank shift such as structures in the Weberian apparatus and haemal arch 27. The paraphophyses on the other hand showed a negative rank shift. In the appendicular skeleton (55 elements examined in total), 28 elements showed a forward rank shift which are associated with swimming such as the anal and dorsal proximal radials and haemal spines of preural centra 2 and 3. The difference in the order of appearance of cartilage structures based on CF50*_lgt_* values between trained and control fish was similar to the difference in the order of appearance of cartilage structures based on CF50*_age_* values between trained and control fish (data not shown).

### Effect of Swim-training on Osteogenesis

The onset of osteogenesis for each bone structure (trained and control fish) was estimated by the age or body length at which 50% of the fish showed the presence of a bone structure. We refer to the estimates of age as BF50*_age_* (Bone Forming) and to the estimates of body length as BF50*_lgt_*. BF50*_age_* and BF50*_lgt_* values were calculated from logistic regressions of the binary presence or absence data of bone structures (see also Materials and Methods). Plotting the BF50*_age_* values of the trained fish against the BF50*_age_* values of control fish showed that swim-training also accelerated osteogenesis (Figure 2E). Most of the BF50*_age_* values were located below the reference line, which indicates that bone structures appeared earlier in trained fish. The difference in BF50*_age_* values between trained and control fish increased with age. This difference in BF50*_age_* values between control and trained fish increased to more than 1 day at 12 dpf and onwards. Swim-training accelerated both intramembranous and perichondral ossification. However, plotting the BF50*_lgt_* values of trained fish against the BF50*_lgt_* values of control fish showed that trained fish were slightly longer at the onset of osteogenesis (Figure 2F, P<0.0001).

**Figure 5:**
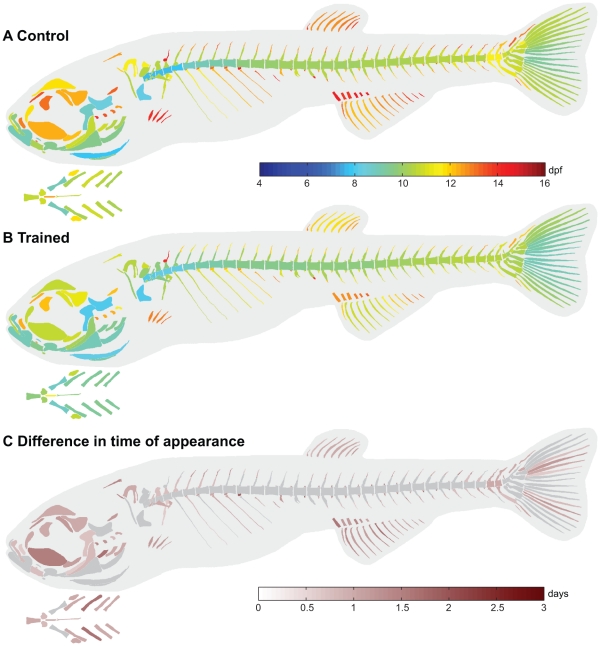
Swim-training had a differential effect on on the age at appearance of bone structures between control and trained fish. **A,B**) BF50*_age_* values visualized in the corresponding structures in control fish (A) and trained fish (B). The branchial region is indicated separately, ventral view. **C**) Differences in BF50*_age_* values between control and trained fish. Positive values indicate that structures appear earlier in the trained fish. Structures with a difference less than twice the standard error are indicated in grey.

In the control fish, some structures already appear before 5 dpf (e.g. cleithrum, opercle and parasphenoid (Figure S2))and the BF50*_age_* values of these structures could therefore not be calculated from our data. The bone structures to ossify in the control fish between 5–8 dpf were the anterior-most 5 centra and branchiostegal ray 2 (Figure 5A and Figure S2). Osteogenesis proceeded (8–10 dpf) with the ossification of the first caudal finrays, centra 6–12 and structures in the cranial skeleton (maxillary, dentary, quadrate, entopterygoid, hyomandibular, pro-otic, ceratohyal, branchiostegalray 3, interopercle, subopercle and basioccipital). The following structures ossified between 10–12 dpf in the axial skeleton: structures of the Weberian apparatus (e.g. claustrum, intercalarium, tripus), centra 13–28, ribs 4–7, the haemal arches (14–28) and spines (18,20–21), neural arches (3–28) and postzygapophyses (pstz, 15–18), many structures of the caudal peduncle (e.g. (pre)urals, hypurals, spines and arches on the preural vertebrae 2 and 3) and the caudal finrays. In the cranial skeleton, the following structures ossified between 10–12 dpf: premaxillary, lateral ethmoid, frontal, anguloarticular, retro-articular, sympletic, preopercle, basihyal, urohyal, ventral hypohyal, epihyal, ceratobranchials 1–4, posttemporal, exoccipital and supracleithrum. The following structures ossified between 12–14 dpf in the axial skeleton: parapophyses (pop, 5–9), ribs (8–11), haemal arch (13) and spines (16–17, 19, 22–28), neural spines (5–28), haemal (14–28) and neural pstz (6–14, 19–28), anal fin rays (5–11), dorsal finrays (1–8), anal proximal radials (apr, 2,3), neural spines of pu 2 and 3 and their respective caudal finrays. In the cranial skeleton, the following structures ossified between 12–14 dpf: infraorbital 1, supraorbital, orbitosphenoid, pterosphenoid, sphenotic, pterotic, supraoccipital, ectopterygoid, metapterygoid, coronomeckelian, basibranchial and epibranchial 2–4. The bones which appeared at 14 dpf and later were epibranchial 1, neural spine 4, pop 10, rib 12, haemal spine 15, neural pstz 2, 5, haemal pstz 13, pectoral finrays, apr 1, 4–6 and anal finrays 12–13.

Analysis of the difference in BF50*_age_* values (temporal shift) between control and trained fish showed that swim training had a differential effect on the temporal shift of bone structures (Figure 5C). The average standard error of all BF50*_age_* values in the trained and control fish was similar (0.22 days). The minimum and maximum standard errors were also similar (control: min 0.20 days, max 0.26 days; trained: min 0.20 days, max 0.25 days). Of the 292 bone elements analyzed in total, 186 showed a temporal shift larger than 2x SED. The minimum temporal shift of these bone elements was 0.64 days, the maximum temporal shift was 2.00 days. On average osteogenesis in these elements occurred 1.1 days earlier in the trained fish. In the cranial skeleton (45 elements examined in total), 32 elements had a temporal shift larger than 2x SED such as the ceratobranchials 1–4, epibranchials 1–4, hyomandibular, maxillary and frontal. In the axial skeleton (171 elements examined in total), 99 elements had a temporal shift larger than 2x SED such as structures in the Weberian apparatus, ribs and paraphophyses, hemal and neural spines, hemal and neural pstz, na (3–8, 27–28) and ha (13, 26–28). In the appendicular skeleton (76 elements examined in total), 55 elements had a temporal shift larger than 2x SED such as the anal proximal radials and fin rays, dorsal finrays, pectoral finrays, structures in the caudal peduncle and caudal finrays.

Analysis of the difference in order of appearance (rank shift, based on BF50*_age_*) of bone structures between control and trained fish showed that swim training had also a differential effect on the rank shift of bone structures (Figure 4B). Of the 292 bone elements analyzed in total, 137 elements showed a forward rank shift in the order of appearance in the trained fish versus the control fish. In the cranial skeleton (45 elements examined in total), 13 of the 20 elements which showed a forward rank shift are associated with ventilation and feeding such as the ceratobranchials 1–4, opercular bones and hymandibular. Elements in the neurocranium and lower jaw on the other hand showed a negative rank shift. In the axial skeleton (171 elements examined in total), 62 elements showed a forward rank shift and are associated among others with the elevation of the head such as the first 6 neural arches in the precaudal skeleton or in the articulation between the vertebrae such as the zygapophyses. The ribs and centra, however, showed a negative rank shift. In the appendicular skeleton (76 elements examined in total), 55 elements showed a forward rank shift and are associated with swimming such as the anal and caudal finrays. The difference in the order of appearance of bone structures based on BF50*_lgt_* values between trained and control fish was similar to the difference in the order of appearance of bone structures based on BF50*_age_* values between trained and control fish (data not shown).

## Discussion

Our study is the first one to show that swim-training increased growth already during early larval development in zebrafish (Figure 1). Previous work from our lab showed that swim-training transiently increased growth during late larval and juvenile development in zebrafish [18] and Palstra *et al.* [26] recently demonstrated that exercise training also increased growth in adult zebrafish. Furthermore, previous work on training in salmonids has shown that exercise at low swimming speeds (<1.5 BL/s) increased growth (reviewed by Davison [27]). The increased growth was related to an increased food intake and a better food conversion efficiency. Our results are, however, in contrast to the study performed by Bagatto *et al.* [28], where swim-training did not affect growth during early development. This could be due to the lower exercise intensity employed in our study. Mortality was also lower in our study. Bagatto *et al.* [28] argued that the high mortality was due to a suboptimal feeding regime during which larvae were not able to acquire sufficient *Paramecium* or *Artemia* to survive (especially the first day of) training. In our swim-training experiments, we overcame this problem by supplementing the diet of the larvae with Liquifry (a milky suspension of yeast and proteins) which increased survival in earlier pilot experiments. In summary, our study demonstrated that swim-training can already increase growth during early larval development if the training and feeding regime are carefully chosen.

In our study, skeletal development in the control fish was as reported by Cubbage and Mabee [5] and Bird and Mabee [6]. The first structures to develop in the cranial skeleton were associated with feeding and respiration. In the axial skeleton, the first regions to develop were the Weberian region and the caudal fin region. Thus, similar to the sucker *Catostomus macrocheilus*, structures which formed earliest were associated with movement rather than protection [3].

Swim training induced an earlier onset of both chondrogenesis and osteogenesis in trained fish (Figure 2). Interestingly, the difference in CF50*_age_* values (temporal shift) of cartilage structures between control and trained fish was close to constant over the range of ages investigated whereas the temporal shift of bone structures between control and trained fish became more pronounced in later stages. Cloutier *et al.* [17] also found that early forming bone elements were less responsive than late forming bone elements. The following two hypotheses can explain this observation: 1) early forming bones are more developmentally constrained than late forming bones and the latter have a higher degree of plasticity [29]–[30] or 2) the time of exposure to the training regime is longer for the late forming bones, which possibly leads to larger cumulative effects on development. However, we do not observe similar effects on the formation of the cartilage structures. This suggests that swim-training has a different regulatory effect on the development of cartilage than on bone structures. Furthermore, swim-training accelerated both the onset of bone formation via perichondral ossification and via intramembranous ossification (based on CF50*_age_* and BF50*_age_*). In addition, the duration of the cartilage phase (the phase from the onset of chondrification until the onset of ossification) of perichondral bones was not affected by swim-training. This indicates that although the onset of chondrogenesis and osteogenesis in these bones was shifted forward due to the swim-training, the duration of the cartilage phase is rather fixed.

In zebrafish, skeletal development is best correlated with size [5]–[6] [31]. As mentioned above, swim-training increased growth in trained fish and accelerated chondrogenesis and osteogenesis. The analysis of the difference in CF50*_lgt_* and BF50*_lgt_* values between trained and control fish, however, showed that trained fish were longer than control fish at the onset of chondrogenesis and osteogenesis. These results suggest that swim-training accelerated overall larval development but growth was accelerated more than chondrogenesis and osteogenesis.

The main goal of our study was to investigate the role of mechanical loading (via swim-training) in prioritizing the development of structures involved in feeding, respiration and swimming. We therefore also analyzed the difference in order of appearance which showed if swim-training lead to a shift in priorities during chondrogenesis and osteogenesis between control and trained fish. Furthermore, this difference in order of appearance gave similar results either with the order of appearance based on the length or on age at appearance of cartilage and bone structures. The results discussed in the next paragraphs are based on the difference in order of appearance of cartilage and bone structures between control and trained fish.

We found that the development of skeletal elements associated with feeding and respiration in the cranial skeleton was prioritized during osteogenesis in trained fish (Figure 4). Interestingly, the development of many elements of the neurocranium was not prioritized. The skull of the zebrafish is a complex structure compared to the mammalian skull regarding the number of bones and articulations [32] [5]. Many of the bones in the splanchocranial and opercular region are involved in respiration and feeding (e.g. hyomandibular, operculars, branchial arches, hyoid arch) [3] [5] [4]. The onset of chondrification and ossification of the skeletal elements involved in feeding and respiration takes place in the first two weeks post fertilization. In this period, zebrafish larvae also make the transition from cutaneous respiration to gill respiration [33]. At 14 dpf, gills are required for O_2_ uptake and ionoregulation [33]. In fish, ventilation takes place by actively pumping (via the muscular pump of the buccal cavity) water across the gills filaments [32]. The increased swimming activity by trained fish presumably increased the oxygen uptake leading to an increase in ventilation. Ballintijn [34] showed in carp that during high intensity breathing more muscles are active than during normal breathing. This suggests that an increase in ventilation due to swim-training increased muscle induced mechanical forces on cranial bones associated with respiration.

Besides those bones in the cranial skeleton that are associated with feeding, the development of bones in the dorsal part of the precaudal axial skeleton (Figure 4; neural arches 3–8) was also prioritized during osteogenesis in trained fish. In zebrafish larvae, a suction feeding event commences with the opening of the mouth by elevation and protraction of the hyoid by the protractor hyoideus and elevation of the head by the epaxial musculature [35]. The epaxial musculature attaches to the dorsal structures of the axial skeleton and inserts on the posterior aspect of the neurocranium (e.g. supraoccipital). The head lift and mouth opening are followed by rapid depression of the hyoid by the sternohyoideus. This muscle inserts on the hyoid via a long tendon which later ossifies into the urohyal [35]. The higher swimming activity in trained fish presumably increased the food uptake which possibly augmented the mechanical loads on these structures. Thus, this suggests that mechanical load induced by muscle forces plays an important role in prioritizing the development of the skeletal elements associated with feeding.

The formation of several elements of the Weberian apparatus was prioritized during chondrogenesis and osteogenesis (e.g. roofing cartilage, tripus, na 3 and 4, Figure 4). The Weberian apparatus plays an important role in the transmission of vibrations from the swim bladder to the inner ear [6] [36]. It is not known if the Weberian apparatus also has a direct role in feeding, respiration or swimming [6] [36]. Furthermore, it is also puzzling why the formation of only certain structures such as the roofing cartilage, tripus, na3 and 4 was prioritized but not the formation of the centra 1–4, scaphium and claustrum. Analysis of the distribution of mechanical load in this region during swim-training might explain the shift in priorities during skeletal development of the Weberian apparatus.

Interestingly, in the axial skeleton, the development of the postzygaphophyses was prioritized during osteogenesis in trained fish but not the development of the centra (Figure 4). Furthermore, the development of the ribs, which provide protection of the various organs in the visceral cavity, was also not prioritized. The axial musculature of fishes supplies the major propulsive forces for locomotion and attaches to the vertebral column [32]. It was therefore expected that the increased swimming activity would also prioritize the development of the centra. These results suggest that postzygaphophys and elements in the cranial and appendicular skeleton experienced different mechanical loads than the centra [37], which prioritized the formation of those elements over the formation of the centra. During the swim-training, the notochord develops into a vertebral column with stiff vertebrae linked by intervertebral ligaments. Zygaphophyses play an important role in the articulation between successive vertebrae and increase the stability of the vertebral column [32] [6]. They form at the posterior ends of centra and are attached with ligaments to the prezygaphophyses of the immediately posterior vertebra [6]. The development of stiff vertebrae in later stages could have altered the distribution of the mechanical load along the axial skeleton with high peak loads in the intervertebral joints. Furthermore, the swimming performance of zebrafish larvae also improves during ontogeny [38]. This might have increased the mechanical loads specifically on the postzygaphophyses during swim-training and thus prioritized their formation.

Moreover, the development of structures in the median fins was prioritized during chondrogenesis and osteogenesis in trained fish (Figure 4). Cloutier *et al.* [17] investigated the effect of swim-training on the onset of skeletogenesis in the median fins in the Arctic charr (*Salvelinus alpinus*). They also found that an increased swimming activity accelerated chondrogenesis and osteogenesis in the median fins. The median fins are flexible and are used for propulsion, braking and steering [39]. The posterior part of the caudal axial skeleton, the caudal peduncle, is modified to transmit forces generated by the axial muscles to the tail fin [39] [18]. The development of the median fins improves swimming performance by increasing the lateral body profile which increases thrust during swimming [40]–[41]. The increased swimming activity increased the activity of the axial musculature which presumably increased the loads on skeletal elements of the median fins. This implies that an increased swimming activity prioritized the formation of skeletal elements in the median fins which probably improved swimming performance.

As stated in the Introduction, it has been shown that fish larvae show the fastest growth in the head and tail area [2] [1]. Furthermore, the skeletal structures which form earliest are in the head and tail area and are essential for the survival of the fish larvae. These structures are involved in feeding, respiration and swimming. Interestingly, in our study, not only the development of elements in the head and tail area was prioritized during chondrogenesis and osteogenesis but also the development of elements in the anal and dorsal fins and axial skeleton. This suggests that an increased swimming activity gave priority to structures which play an important role in swimming and thereby increasing the chance of survival in an environment with a higher water velocity.

Our study is the first to show that during early zebrafish larval development, skeletal tissue in the cranial, axial and appendicular skeleton is competent to respond to swim-training due to increased water velocities [17] [22]–[25]. It demonstrates that changes in water flow conditions can result into significant spatio-temporal changes in skeletogenesis. Since the zebrafish as a model system enables a diverse range of genetic analyses as well as possibilities to investigate development on a cellular, tissue and organ level, our study might facilitate in the future the connection of information about environmental influences with altered gene expression profiles and the resulting morphological changes.

## Materials and Methods

### Husbandry

Wildtype zebrafish were reared at the fish facility of Wageningen University at 27.1°C under standard conditions. Eggs were collected by mating two males with three females. Eggs were kept at 28.5°C at the breeding facility, and transferred to the fish facility at 3 dpf.

### Ethics Statement

The swim-training experiments were approved by the Wageningen University Animal Experiments Committee (protocol nr. 2009046f).

### Swim-training Setup

**Figure 6:**
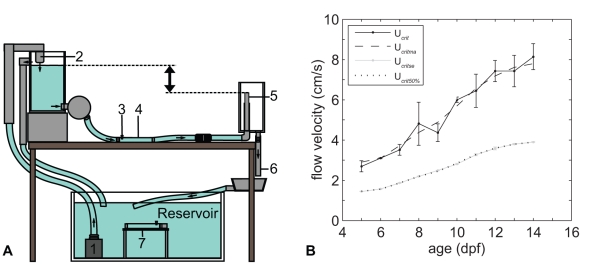
Schematic representation of the swim-training set-up in lateral view and critical flow velocity. **A**) Water was pumped (1) to the top aquarium and flowed back into the reservoir via the training tubes (4), outflow tubes (5) and outflow hoses (6) due to gravity. The difference in water level between the top aquarium and the outflow tubes (5) (indicated with up down black arrow) determined the flow velocity in the training tubes (4). Control fish were kept in similar tubes in the same set-up (7). Both the training and control section consisted of five tubes placed parallel to each other (not visible in drawing). Each tube had its own outflow tube and hose. **B**) Critical flow velocity (*U_crit_*) over time and during swim-training experiments (*U_critse_*). Zebrafish were subjected to 50% (*U_crit_*_50%_) of the moving average *U_crit_* (*U_critma_*).

The swim-training setup is a gravity-fed system (Figure 6A) and is located in a temperature controlled room. The part of the tubes in which the fish were kept had an internal diameter of 2 cm and a length of 20 cm for a total volume of 62.8 ml water. Fish were retained in the tubes by fine nylon mesh (0.4 mm x0.4 mm, from core to core of the wires) on each end of the tube. A parallel array of tightly packed straws (inner diameter of 6.13 mm) was placed before the first nylon mesh to create laminar flow in the tube. The water in the swim-training set-up was copper free and filtered with a biological filter system (Eheim). Water samples were taken daily from a random tube (control and trained) to measure the water quality (ammonia, nitrite, nitrate, pH and conductivity). Ammonia, nitrite and nitrate did not reach toxic levels during the swim-training (ammonia/nitrate<0.03 mg/ml, nitrate<5 mg/ml). pH (7.74 ± 0.03) and conductivity (884.86 ± 7.78 µS/cm) also remained within the range of standard conditions. Water temperature (28.4 ± 0.16°C) was measured daily.

The flow velocity was calculated by dividing the flow rate by the cross sectional area of the tube and thus represents the average flow velocity throughout the article unless otherwise stated. The difference in water level between the top aquarium and the outflow tubes determined the flow velocity in the training tubes (up down black arrow, Figure 6A). Thus, by adjusting the height of the outflow tubes, the flow velocity could be adjusted in the training tubes. Each training tube had its own outflow tube which allowed for fine adjustment of the flow velocity in each training tube. PIV (Particle Image Velocimetry) analysis of the water velocity in the tubes showed that at the entrance of the tube the boundary layer was thin and the flow had a nearly uniform velocity over the cross-section. Near the outflow of the tube, the flow profile had developed into a Hagan-Poisseuille flow profile (Figure S3). Furthermore, the higher the flow velocity, the more time it took to fully develop a Hagan-Poisseuille flow profile.

### Training Regime

We first determined the critical swimming speed of zebrafish larvae from 5–14 dpf using the method developed by Brett [42]. The critical swimming speed is a standard measurement to assess the aerobic swimming performance of fish [42]–[43]. In this method, fish were subjected to intervals of swimming (for a prescribed duration) at a certain flow velocity. After each interval, the flow velocity was increased at a prescribed increment. After several intervals and increments, fish displayed symptoms of severe fatigue, did not swim anymore and were eventually swept against a mesh, indicating that fish had reached their critical flow velocity. The critical flow velocity is calculated with the following equation [42]:
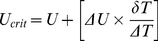
(1)where *U* = flow velocity at the last interval where fish swam the whole interval (cm/s), 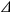 *U* = velocity increment (cm/s), 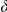 *T* = time that fish swam in the last interval (seconds), 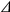 *T* = duration of one interval (15 min). Zebrafish larvae (n = 10) were placed in a training tube and allowed to acclimate for half an hour. In the first interval of 15 min, fish were subjected to a flow velocity of 1.15 cm/s (3 body lengths per second (Bl/s), 5 dpf) to 1.91 cm/s (3 Bl/s, 14 dpf). In the next 15 min intervals, flow velocity was increased with increments of 1 Bl/s (4–7 dpf) or 2 Bl/s (8–14 dpf). After several intervals and increments, fish reached their critical flow velocity. The critical flow velocity (*U_crit_*) was defined as that velocity when 50% of the fish were against the mesh at the end of the tube or held position to the wall for more than 3 seconds. These experiments were performed *in duplo* (morning and afternoon) from 5–14 dpf (Table 1, *U_crit_* I and II). The critical flow velocity with standard deviation was plotted over time in Matlab (Figure 6B, *U_crit_*). To smooth out the data and to highlight the trend we calculated the moving average of the critical flow velocity of 5–14 dpf (Figure 6B, *U_critma_*). Zebrafish larvae were subjected to 50% of this *U_critma_* during the subsequent experiments (Figure 6B, *U_crit_*_50%_ and *U_critse_*). Note that since especially the young larvae adhere to the wall of the tube, or swim close to the wall in the boundary layer, the measured critical swimming speed cannot be interpreted as the actual water velocity in which the larvae can just hold position. Our measured critical swimming speed is an overestimation of the maximum sustained swimming speed.

**Table 1:** Number of fish sampled during each stage in a swim-experiment.

swim-training experiment	5 dpf	6 dpf	7 dpf	8 dpf	9 dpf	10 dpf	11 dpf	12 dpf	13 dpf	14 dpf
Ucrit I (morning)	10	10	10	10	10	10	10	10	10	10
Ucrit II (afternoon)	10	10	10	10	10	10	10	10	10	10
Cartilage I (control)	4	5	4	4	5	5	4	4	4	5
Cartilage I (trained)	3	3	3	4	1	2	3	3	4	4
Cartilage II (control)	5	4	4	3	2	3	3	2	4	3
Cartilage II (trained)	4	4	4	5	4	5	5	5	5	5
Bone I (control)	1	2	2	3	2	3	3	5	4	5
Bone I (trained)	5	5	5	4	5	5	4	4	5	5
Bone II (control)	5	4	4	5	5	5	5	5	5	5
Bone II (trained)	5	5	5	4	5	5	5	5	5	5

Bagatto *et al.* [28] subjected larval (4–14 dpf) zebrafish to a training regime at night of 2 Bl/s (yolk sac larvae, 4–6 dpf) for 24 hours and 5 Bl/s (swim up larvae, 9–14 dpf) for 15 hours. Bagatto *et al.* [28] showed that this training regime was too severe, especially for the larvae with a yolk sac. To avoid analyzing effects of the swim-training confounded by high mortality we chose for a less severe training regime. Therefore, we chose to subject the zebrafish to 50% of the *U_critma_* (Figure 6B, *U_crit_*_50%_) for 9 hours daily. Furthermore, to be able to monitor the swimming behaviour and the flow velocity, fish were trained during the day. During the swim-training experiments the absolute values corresponded to a relative water velocity of 3.4 ± 0.04 Bl/s at 5 dpf to 5.1 ± 0.6 Bl/s at 14 dpf.

At 4 dpf, 10 fish were randomly assigned to each control and training tube (5 control and 5 trained tubes) in the swim-training set-up. Fish were placed in the swim-training set-up on 4 dpf to acclimate to the conditions. From 5–14 dpf, zebrafish larvae were subjected to 3 training sessions of 3 hours (8∶30–11∶30, 12∶00–15∶00, 15∶30–18∶30) per day. For refreshment there was a continuous water flow of less than 0.1 Bl/s in the control tubes but only when the trained fish were subjected to swim-training. Fish were fed before the first training session and after each training session (during breaks of 30 minutes). Fish were fed *Paramecium* (until 9 dpf), Liquifry no.1 (until 7 dpf, Interpet, Surrey, England) and *Artemia* (from 8–14 dpf, live brine shrimp, Salt Lake Aquafeed, Utah, USA) *ad libitum*. Fish were always fed first *Artemia* and then *Paramecium*. During the swim experiment fish were subjected to a photoperiod regime of 12L:12D. Four swim experiments were performed in total, Cartilage I/II and Bone I/II (Table 1).

### Sampling and Length Measurements

At the end of each training day (18∶30), one fish was sampled randomly from each tube (Table 1, swim training experiments Cartilage I and II, Bone I and II). Subsequently, fish were euthanized with 0.1% tricaine methane sulphonate (TMS, Crescent Research chemicals, USA) buffered with 0.08% sodium bicarbonate (Gibco, Paisley, Scotland). Subsequently, images of sampled fish were taken in the TMS solution to measure the length. Images were taken with an Olympus DP50 digital camera mounted on a Zeiss Stemi SV11 microscope and with AnalySIS*^D^* software (Soft Imaging System GmbH, Germany). After the length measurements, fish were fixed and stored in 4% PFA (paraformaldehyde, Merck, Darmstadt, Germany) in PBS (phosphate buffered saline) at 4°C. Only for the bone staining, fish were transferred the following day to 70% methanol (Merck, Darmstadt, Germany) and stored at 4°C in 70% methanol.

Standard length was measured with the measurements tool in AnalySIS^D^ software of samples from four swim training experiments (Table 1, Cartilage I–II and Bone I–II). Standard length was measured from the anterior end of the upper jaw to the posterior end of the hypurals. In preflexion and early flexion larvae the notochord length was measured from the anterior end of the upper jaw to the posterior tip of the notochord [6]. Standard length measurements of control and trained fish in relation to age were evaluated with quadratic regression for two groups in SAS v9.2, P<0.05 was accepted as significant. Average length measurements over time with standard deviation were plotted in Matlab 7.6.0.324 (R2008a, The Mathworks, Inc.).

### Swimming Behaviour

The swimming behaviour of the larvae in a randomly chosen training tube was observed for half an hour during each training session (3 training session per day, during the swim-training experiments Bone I–II, Table 1). An example of the swimming behaviour of control and trained fish is provided in Movies S1 and S2 of 11 dpf. After each minute during that half an hour the following points were noted: the number of fish swimming, position in the tube (front part or back part), number of fish swimming along the wall of the tube, attached to the wall or against the mesh (for more than 3 seconds). During each break (of half an hour), the number of fish swimming was noted directly after turning the waterflow off and then every following five minutes in a random training tube. The control fish swam in all directions throughout the tube and did not show a specific behaviour such as the trained fish.

### Burst Frequency

To analyze the burst frequency (bursts/s), the swimming behaviour of the trained and control fish was recorded with a Casio EX–F1 camera (with 30 fps in HD (1280 × 720)) during the 3rd training session (13∶00–14∶30) during the swim-training experiment Bone II (Table 1, Movies S1 and S2). The number of bursts were counted of one fish per tube (n = 5, control/trained) during 10 consecutive seconds (300 frames). To accurately count the number of bursts, the play back rate of the movies was decreased to 3 frames per second with the Photron Fastcam viewer 2.4. Average burst frequencies over time with standard deviation were plotted in Matlab 7.6.0.324 (R2008a, The Mathworks, Inc.). Burst frequencies of control and trained fish in relation to age were evaluated with an ANCOVA in SAS 9.2, P<0.05 was accepted as significant.

### Cartilage and Bone Staining

Both the cartilage and bone staining was performed essentially as described previously [44]. Fish from two independent swim-training experiments were stained for cartilage (Table 1, swim-training experiments Cartilage I and II). Fish from one swim-training experiment were stained for bone (Table 1, 8–14 dpf, swim-training experiment Bone II). For the bone staining, the larvae were first rehydrated in 50% methanol, then rinsed in 0.2% Triton X100 (Sigma, St. Louis, USA) and bleached/degreased with 0.9% H_2_O_2_/0.8% KOH in 0.2% Triton X100. Larvae of 5–9 dpf were incubated in the bleach solution for 20 min, the larvae of 10–14 dpf for 30 minutes. Larvae were then neutralized in 100% saturated sodium tetraborate (Di-sodium tetraborate decahydrate, extra pure, Merck, Darmstadt, Germany). To make them more permeable for the alizarin red staining, larvae were digested with 0.1 g/ml trypsin (pancreas protease, Merck, Darmstadt, Germany) in 60% saturated sodium tetraborate/0.2% Triton X100. Larvae of 5–9 dpf were incubated in the digest solution for 30 minutes, larvae of 10–14 dpf for an hour. Bones were stained with 0.04 mg/ml alizarine red (Merck, Darmstadt, Germany) in 1% KOH. Subsequently, larvae were washed and cleared with 20% glycerin/0.8% KOH in 0.2% Triton X100. The larvae were rinsed with 30% glycerin and stored in 70% glycerin/10% KOH solution at 4°C.

For the cartilage staining, larvae were first stained with 0.15 mg/ml Alcian blue (Alcian blue 8GX, Acros Organics, New Jersey, USA) in 30% Acetic Acid/70% ethanol overnight. Larvae were rehydrated in 50% and 30% ethanol and rinsed in 0.2% Triton X100. Larvae were then bleached, neutralized, digested and stored in glycerin at 4°C according to the bone staining protocol. All experiments were performed with demineralized water and at room temperature.

Subsequently, a control or trained fish was chosen randomly and the presence or absence of a cartilage/bone structure was scored within one month. Cartilage structures were scored as present upon visualization of Alcian blue staining of the glycosaminoglycans in cartilage. Bone structures were scored as present upon visualization of Alizarine red staining of calcium in mineralized bone matrix. Images of the bone or cartilage staining of zebrafish larvae were taken with an Olympus DP50 digital camera mounted on a Zeiss Stemi SV11 microscope and with AnalySIS^D^ software (Soft Imaging System GmbH, Germany).

### Statistical Analysis of Cartilage and Bone Formation

The onset of chondrogenesis and osteogenesis for each cartilage and bone structure (trained and control) was estimated by the age or body length at which 50% of the fish showed the presence of a cartilage and bone element. We refer to these estimates of age as CF50*_age_* (Cartilage Forming) or BF50*_age_* (Bone Forming) values and to the estimates of body length to as CF50*_lgt_* or BF50*_lgt_* values. CF50 and BF50 values (of age and body length) were calculated from logistic regressions of the binary presence or absence data of cartilage and bone structures, using the procedure for generalized linear models (GENMOD) of SAS 9.2, employing a binomial distribution and logit link function. In the logistic regression model the probability *p_ij_* of a cartilage or bone structure *i* to have been developed at day or body length *x*, in treatment group *j* (*j* = 1 trained, *j* = 2 control) is modeled as *logit(p_ij_) = a_ij_+bx*. We used separate models for cartilage and bone structures. To keep the models simple but still usable for our purposes (i.e. to estimate CF50 and BF50 values (of age and body length)), we assumed that bone structures developed at the same rate (so have a common slope b), irrespective of the treatment. With the logistic regression models the CF50 of structure *i* in treatment group *j* was estimated as CF50*_ij_* = *−a_ij_/b*, and likewise for BF50. We excluded bones which were already present at 5 dpf in all fishes of a control or trained group, and bones which were not yet present at 14 dpf in all fishes of one of the groups, as it was impossible to calculate the CF50*_age_* or BF50*_age_* values in these cases. These bones were also excluded from the data with the estimates of the onset of chondrogenesis and osteogenesis in body length. The CF50 values (of age and body length) were average values of two swim-training experiments (Table 1, swim-training experiments Cartilage I and II). The BF50 values (of age and body length) were from a single swim-training experiment (Table 1, 8–14 dpf, swim-training experiment Bone II). The CF50 and BF50 values of the trained fish versus control fish were plotted in Matlab 7.6.0.324 (R2008a, The Mathworks, Inc.). The agreement between trained and control fishes for the different CF50 and BF50 variables was tested using the Bradley-Blackwood procedure. Significant results were followed up by a pairwise t-tests to check for systematic differences, and by t-tests of the slope.

To get an impression of the precision of the CF50*_age_* and BF50*_age_* values, we used the delta-method to compute the approximate standard errors (SE) of the CF50*_age_* and BF50*_age_* values. The delta-method can be used in case of non-linear functions of parameters, like we have here (CF50*_ij_  = −a_ij_/b*). We used the variances and covariances of the parameters from the logistic regressions, as calculated by SAS, and imported these into R (version 2.12.2), using a tailor-made program for the delta-method. The standard error of the difference (SED) of CF50_control_ and CF50_trained_ was calculated as
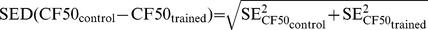
(2)


The standard error of the difference of BF50_control_ and BF50_trained_ was calculated with the similar formula.

To visualize the CF50 and BF50 values with the corresponding cartilage or bone structure, each structure was traced as a separate black structure in a cartilage or bone staining of a 14 dpf trained fish with gimp v2.6. Subsequently, each of these black structures was linked to the corresponding CF50*_age_* or BF50*_age_* value in Matlab 7.6.0.324 (R2008a, The Mathworks, Inc.).

The difference in CF50*_age_* values (hereafter called the temporal shift, in days) between control and trained fish was calculated by subtracting the CF50*_age_* values of the trained fish from the CF50*_age_* values of the control fish. The temporal shift in appearance of bone structures between control and trained fish was calculated similarly using the BF50*_age_* values. This temporal shift between the control and trained fish was visualized with the corresponding cartilage/bone structure with the same method described in the previous paragraph in Matlab 7.6.0.324 (R2008a, The Mathworks, Inc.).

In fish, bones can form via intramembranous or perichondral ossification. During intramembranous ossification, bones form directly from mesenchymal precursors whereas during perichondral ossification, bony matrix forms around a cartilage model [6]. We call the phase from the onset of chondrogenesis until the onset of osteogenesis the cartilage phase. The duration of the cartilage phase in cartilage structures was calculated by subtracting the CF50*_age_* values from the BF50*_age_* values in control and trained fish. The relationship between the duration of the cartilage phase and CF50*_age_* or CF50*_lgt_* values (control and trained) was analyzed with linear regression in SAS 9.2, P<0.05 was accepted as significant.

The order of appearance of the cartilage or bone structures in the control or trained fish was determined by assigning a rank to each cartilage or bone structure (starting with the structure which appeared first in age or body length). The shift in rank for each cartilage and bone structure was determined by subtracting the rank value of the cartilage or bone structure in the trained fish from the rank value of the cartilage or bone structure in the control fish. The shift in rank values were visualized with the corresponding cartilage or bone structure with the same method described above in Matlab 7.6.0.324 (R2008a, The Mathworks, Inc.).

## Supporting Information

Figure S1 **The cartilage structures, indicated here in black, which were analyzed in this study.** A) Alcian blue staining of 14 dpf trained fish, lateral view. The areas indicated in red are shown enlarged in B, C and D. adr, anal distal radial; apr, anal proximal radial; ddr, dorsal distal radials; dpr, dorsal proximal radial; edz1, endoskeletal disc with cartilage subdivision zone 1; ha27/28, haemal arch 27/28; hs, haemal spine 28; na27/28, neural arch 27/28; pop, paraphophysis; ts, posterior end of the tectum synoticum. B) Branchial structures, lateral view. bb, basibranchial; ep, epibranchial; hb, hypobranchial; pb, pharyngobranchial. C) Weberian apparatus, lateral view. in, intercalarium; lp2, lateral process 2; na, neural arch; pop, paraphophysis; rc, roofing cartilage; sc, scaphium; sn, supraneural; tr, tripus. D) Caudal fin, lateral view. ep, epural; hapu, haemal arch of preural; hspu, haemal spine of preural; hy, hypural; napu, neural arch of preural; nspu, neural spine of preural; opstc, opistural cartilage; phy, parhypural. Nomenclature follows Cubbage and Mabee [5], Bird and Mabee [6] and Bensimon-Brito *et al.* [45].
(TIF)

Figure S2 **The bone structures, indicated in black, which were analyzed in this study.** A) Alizarin red staining of 14 dpf trained fish, lateral view. The areas indicated in red are shown enlarged in B, C and D. ha, haemal spine; hs, haemal spine; na, neural arch; ns, neural spine; pf, pectoral finrays; pop, paraphophysis. The haemal postzygaphophysis is marked by a pound sign and the neural postzygaphophysis by an asterisk. B) Cranial skeleton, lateral view. a, anguloarticular; br, branchiostegalray; cb, ceratobranchial; d, dentary; en, entopterygoid; ep, epibranchial; f, frontal; hy, hyomandibular; infra1, infraorbital 1; iop, interopercle; le, lateral ethmoid; m, maxilla; mpt, metapterygoid; os, orbitosphenoid; p, preopercle; pm, premaxilla; pro, pro-otic; pt, posttemporal; pto, pterotic; pts, pterysphenoid; q, quadrate; ra, retroarticular; so, supraorbital; soc, supraoccipital; sop, subopercle; sph, sphenotic; sy, sympletic. The coronomeckelian is marked by an asterisk and the ectopterygoid by a pound sign. C) Weberian apparatus, lateral view. c1/4, centrum 1/4; cl, claustrum; boc, basioccipital (posterior region); eoc, exoccipital; in, intercalarium; lp1/2, lateral process 1/2; na, neural arch; ns, neural spine; os, os suspensorium; pop, paraphophysis; sc, scaphium; scl,supracleithrum; tr, tripus. The neural postzygaphophysis is marked by an asterisk. D) Caudal fin, lateral view. ep, epural; hapu, haemal arch of preural; hspu, haemal spine of preural; hy, hypural; napu, neural arch of preural; nspu, neural spine; pls, pleurostyle; phy, parhypural; pu, preural; u, ural. The neural arch of the urostyle is marked by a triangle, the neural postzygaphophysis of preural 2 by a diamond, the parhypuraphophysis by an asterisk and ural 2 by a pound sign. E) Cranial skeleton, ventral view. bb1, basibranchial; bh, basihyal; cb, ceratobranchial; ch, ceratohyal; eh, epihyal; hhv, ventral hypohyal; uh, urohyal. Nomenclature follows Cubbage and Mabee [5] and Bird and Mabee [6].
(TIF)

Figure S3 **Flow profile in training tubes.** Visualization of the flow profile with Particle Image Velocimetry at different cross sections in the tube. Black dots show the flow profile at the inflow of the tube, dark grey in the middle of the tube and light grey at the outflow of the tube. A) average flow velocity 5.30 cm/s, B) average flow velocity 2.65 cm/s, C) average flow velocity 1.77 cm/s.
(EPS)

Movie S1 **Swimming behaviour of control fish at 11 dpf.** See also Materials and Methods for information about the recordings and analysis.
(MOV)

Movie S2 **Swimming behaviour of trained fish at 11 dpf.** See also Materials and Methods for information about the recordings and analysis.
(MOV)
