# Supplementary figures and images for: Swim-Training Changes the Spatio-Temporal Dynamics of Skeletogenesis in Zebrafish Larvae (Danio rerio)
Source: PLoS One. 2012 Apr 18;7(4):e34072. doi: 10.1371/journal.pone.0034072 (PMC3329525; doi:10.1371/journal.pone.0034072)

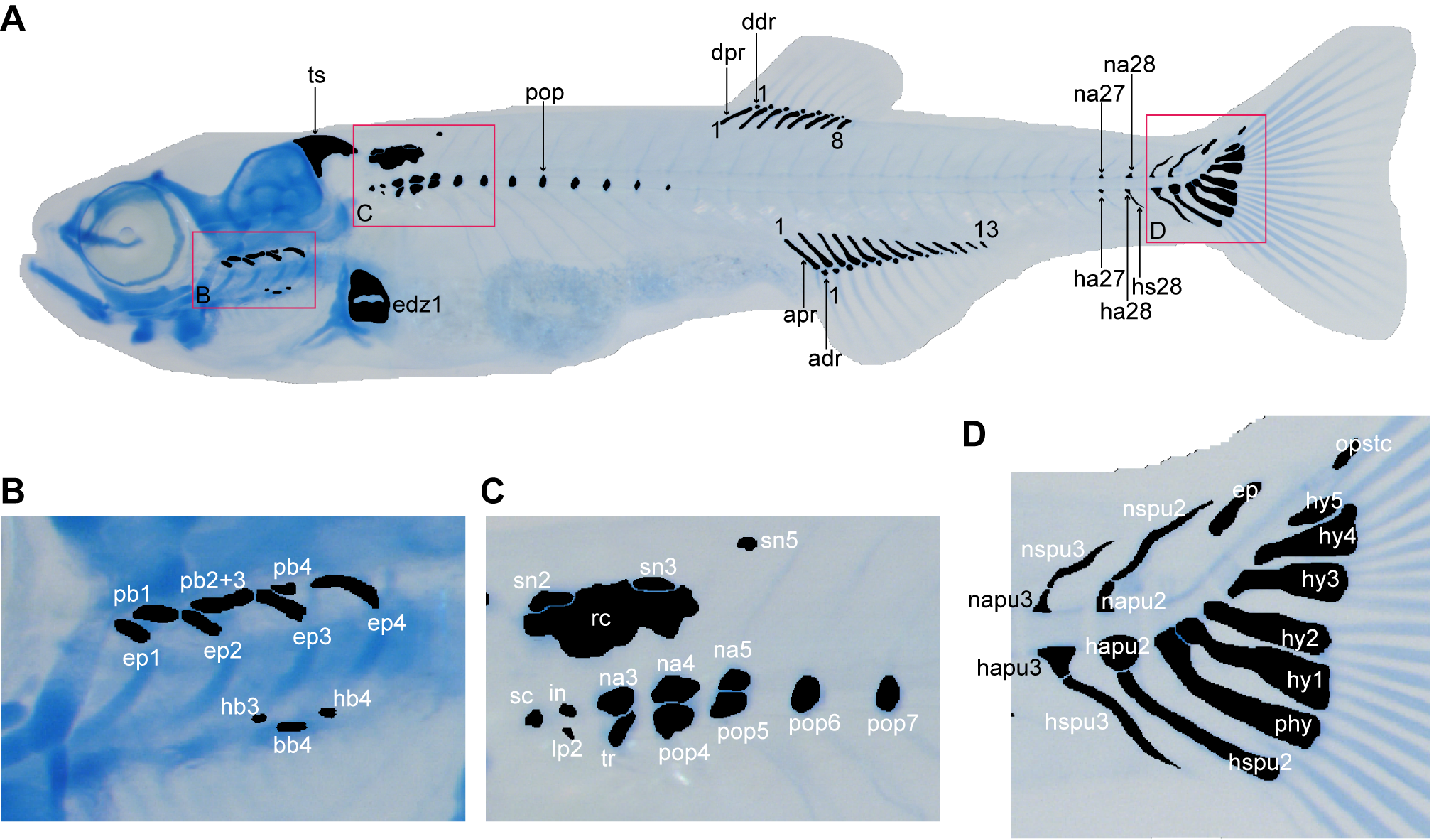

Supplement: Figure S1 — The cartilage structures, indicated here in black, which were analyzed in this study. A) Alcian blue staining of 14 dpf trained fish, lateral view. The areas indicated in red are shown enlarged in B, C and D. adr, anal distal radial; apr, anal proximal radial; ddr, dorsal distal radials; dpr, dorsal proximal radial; edz1, endoskeletal disc with cartilage subdivision zone 1; ha27/28, haemal arch 27/28; hs, haemal spine 28; na27/28, neural arch 27/28; pop, paraphophysis; ts, posterior end of the tectum synoticum. B) Branchial structures, lateral view. bb, basibranchial; ep, epibranchial; hb, hypobranchial; pb, pharyngobranchial. C) Weberian apparatus, lateral view. in, intercalarium; lp2, lateral process 2; na, neural arch; pop, paraphophysis; rc, roofing cartilage; sc, scaphium; sn, supraneural; tr, tripus. D) Caudal fin, lateral view. ep, epural; hapu, haemal arch of preural; hspu, haemal spine of preural; hy, hypural; napu, neural arch of preural; nspu, neural spine of preural; opstc, opistural cartilage; phy, parhypural. Nomenclature follows Cubbage and Mabee [5], Bird and Mabee [6] and Bensimon-Brito et al. [45]. (TIF) [file pone.0034072.s001.tif]

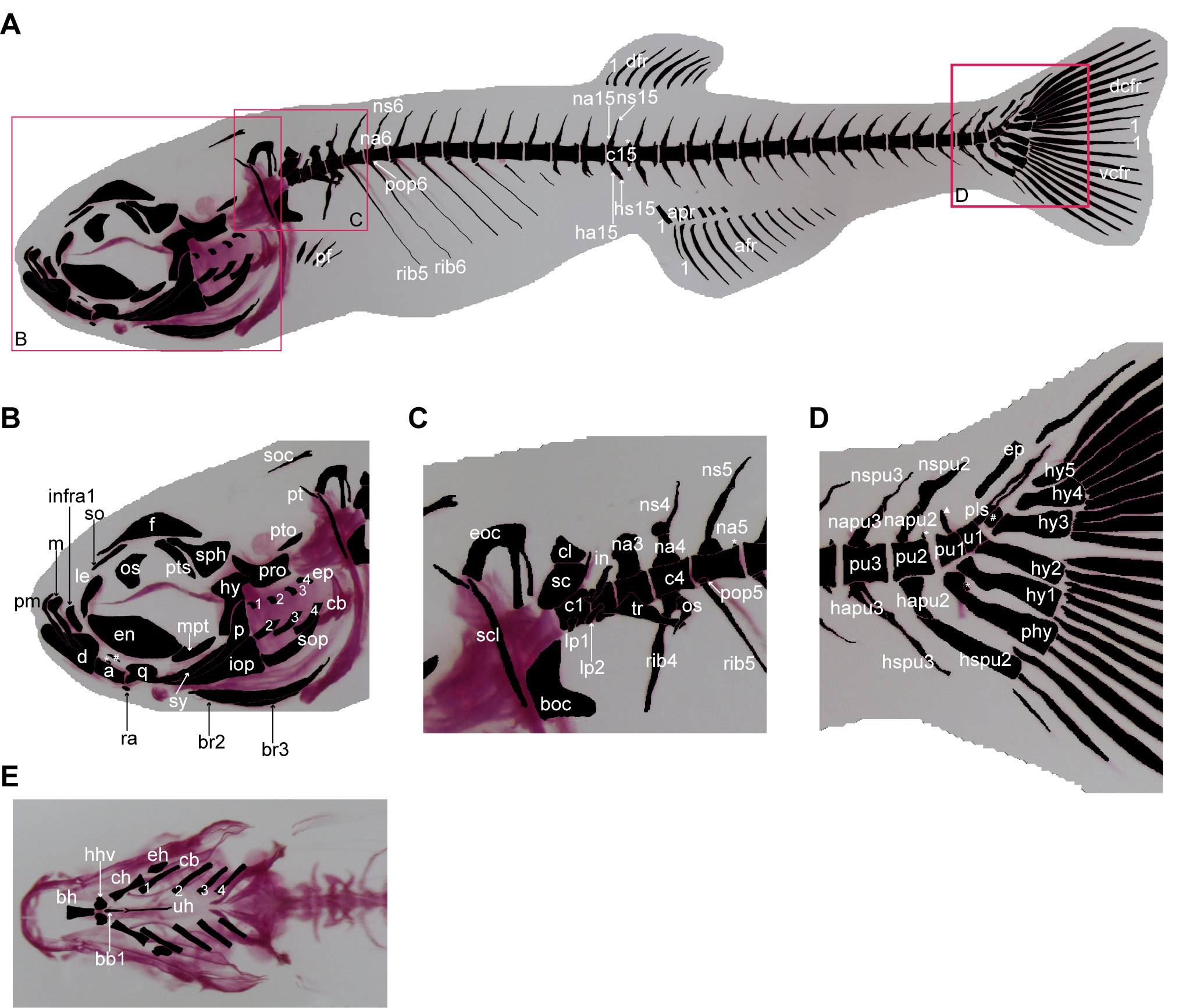

Supplement: Figure S2 — The bone structures, indicated in black, which were analyzed in this study. A) Alizarin red staining of 14 dpf trained fish, lateral view. The areas indicated in red are shown enlarged in B, C and D. ha, haemal spine; hs, haemal spine; na, neural arch; ns, neural spine; pf, pectoral finrays; pop, paraphophysis. The haemal postzygaphophysis is marked by a pound sign and the neural postzygaphophysis by an asterisk. B) Cranial skeleton, lateral view. a, anguloarticular; br, branchiostegalray; cb, ceratobranchial; d, dentary; en, entopterygoid; ep, epibranchial; f, frontal; hy, hyomandibular; infra1, infraorbital 1; iop, interopercle; le, lateral ethmoid; m, maxilla; mpt, metapterygoid; os, orbitosphenoid; p, preopercle; pm, premaxilla; pro, pro-otic; pt, posttemporal; pto, pterotic; pts, pterysphenoid; q, quadrate; ra, retroarticular; so, supraorbital; soc, supraoccipital; sop, subopercle; sph, sphenotic; sy, sympletic. The coronomeckelian is marked by an asterisk and the ectopterygoid by a pound sign. C) Weberian apparatus, lateral view. c1/4, centrum 1/4; cl, claustrum; boc, basioccipital (posterior region); eoc, exoccipital; in, intercalarium; lp1/2, lateral process 1/2; na, neural arch; ns, neural spine; os, os suspensorium; pop, paraphophysis; sc, scaphium; scl,supracleithrum; tr, tripus. The neural postzygaphophysis is marked by an asterisk. D) Caudal fin, lateral view. ep, epural; hapu, haemal arch of preural; hspu, haemal spine of preural; hy, hypural; napu, neural arch of preural; nspu, neural spine; pls, pleurostyle; phy, parhypural; pu, preural; u, ural. The neural arch of the urostyle is marked by a triangle, the neural postzygaphophysis of preural 2 by a diamond, the parhypuraphophysis by an asterisk and ural 2 by a pound sign. E) Cranial skeleton, ventral view. bb1, basibranchial; bh, basihyal; cb, ceratobranchial; ch, ceratohyal; eh, epihyal; hhv, ventral hypohyal; uh, urohyal. Nomenclature follows Cubbage and Mabee [5] and Bird and Mabee [6]. (TIF) [file pone.0034072.s002.tif]
